# The Structure of Major Life Transitions Among Older Suicide Decedents: An Application of Large Language Models

**DOI:** 10.1093/geroni/igaf122.1215

**Published:** 2025-12-31

**Authors:** Briana Mezuk, Viktoryia Kalesnikava, David Jurgens, Lily Johns, Kara Zivin

**Affiliations:** University of Michigan, Ann Arbor, Michigan, United States; Institute for Social Research, Ann Arbor, Michigan, United States; University of Michigan, Ann Arbor, Michigan, United States; University of Michigan, Ann Arbor, Michigan, United States; University of Michigan Medical School, Ann Arbor, Michigan, United States

## Abstract

The role of life events in shaping suicide risk for older adults is unclear. Structure and meaning of events are interconnected: structure shapes the relationship between event elements (e.g., timing), with meaning emerging through their dynamic interactions. Large language models (LLM) provide an opportunity to analyze textual data at scale, but they have not yet been widely used to understand the contributing circumstances of suicide. In this study, we applied LLMs to narrative texts from the National Violent Death Reporting System (2003-2022), a nationwide registry of suicide deaths, to (1) identify salient events in the lives of suicide decedents aged 50 + (n = 164,240), (2) classify aspects of the structure of these events (e.g., timing, sequence, expectedness), and (3) explore variation by age. Mean age of decedents was 63 years, 24% were female, 88% were non-Hispanic White; 54% did not have a known mental health problem at the time of their death. Most narratives described sequences of life events involving interlinked health or family problems that often escalated days before suicide (e.g., mobility loss after a recent surgery); less frequently, narratives mentioned isolated events, referring to financial or relationship crises. For decedents aged 65+, events often involved legal issues or relationship loss (e.g., widowhood), while those aged <65 events typically related to employment or relationship discord. Preliminary analyses demonstrate the feasibility of identifying structural elements of life events using LLMs; next steps will include refining prompts to improve classification of complex transitions from these texts.

